# Supplementary material for: Disentangling the interplay of positive and negative selection forces that shaped mitochondrial genomes of Gammarus pisinnus and Gammarus lacustris
Source: R Soc Open Sci. 2020 Jan 8;7(1):190669. doi: 10.1098/rsos.190669 (PMC7029888; doi:10.1098/rsos.190669)
Supplement: Additional file 2. Topologies obtained using different phylogenetic analyses and datasets. [file rsos190669supp2.pdf]

Additional file 2. Topologies obtained using different phylogenetic analyses and datasets.

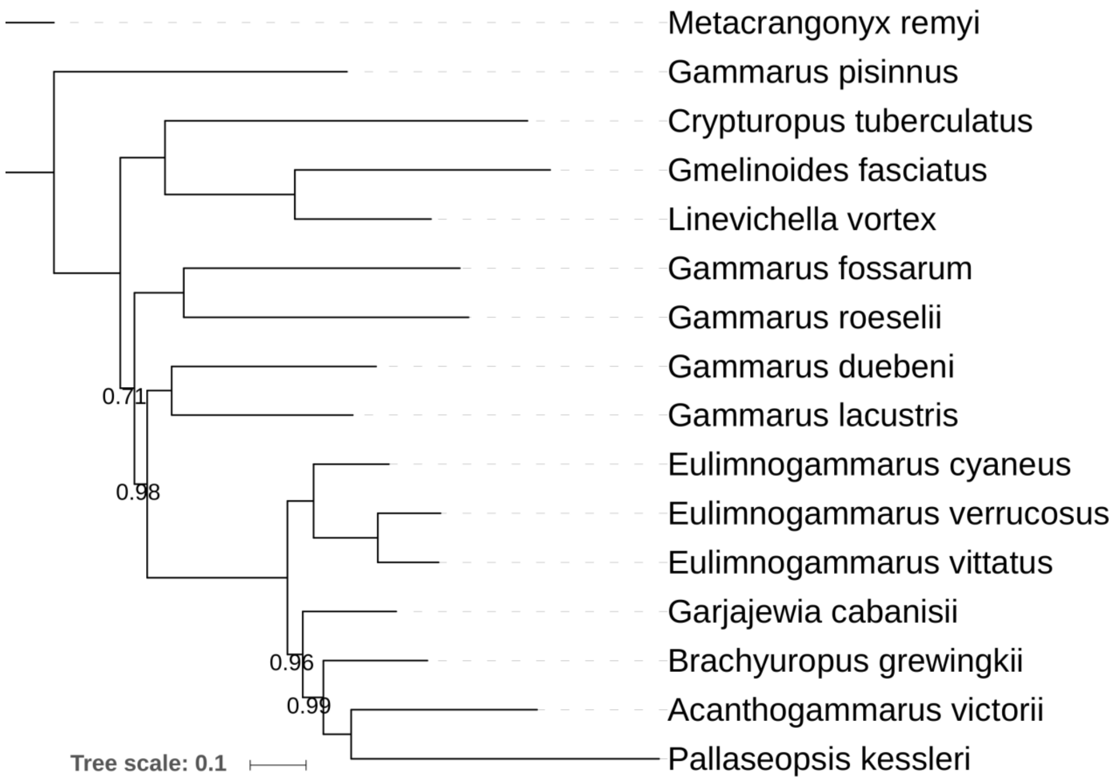

BI_PCG_AA_Part_Gammaroidea analysis. Phylogram was inferred via a Bayesian analysis of concatenated (partitioned) amino acid sequences of all 13 protein-coding genes of the Gammaroidea dataset. *Metacrangonyx remyi* is the outgroup (branch cropped). Scale bar corresponds to the estimated number of substitutions per site. Posterior probability support values <1.0 are shown next to nodes. GenBank accession numbers are available in the Additional file 1.

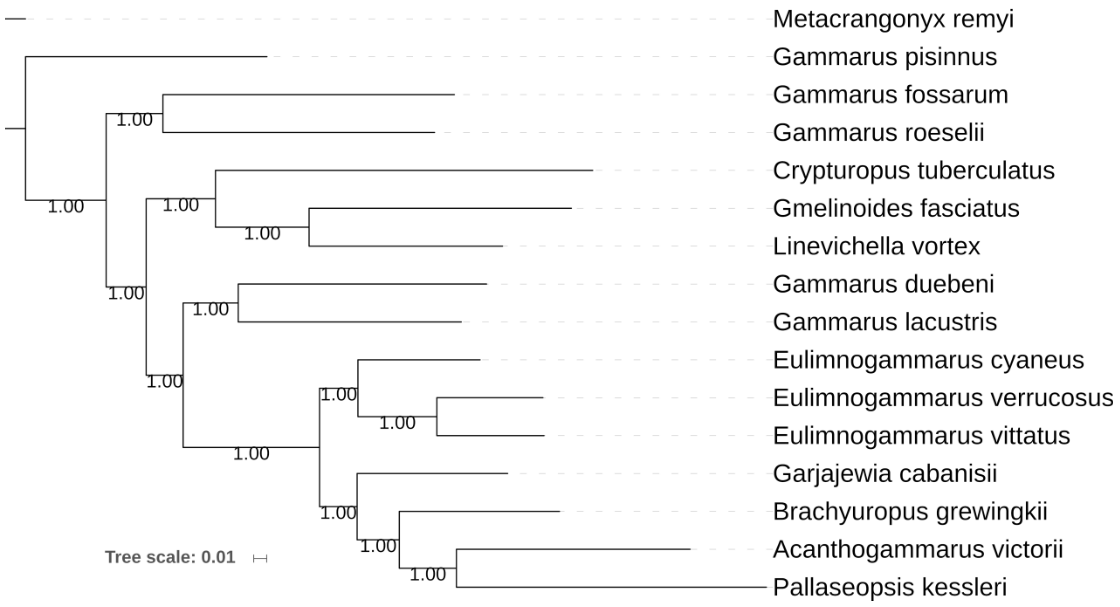

BI_PCGRT_NUC_Part_Gammaroidea analysis. Phylogram was inferred via a Bayesian analysis of concatenated (partitioned) nucleotide sequences of all genes (PCG+rRNA+tRNA) of the Gammaroidea dataset. *Metacrangonyx remyi* is the outgroup (branch cropped). Scale bar corresponds to the estimated number of substitutions per site. Posterior probability support values are shown next to nodes. GenBank accession numbers are available in Additional file 1.

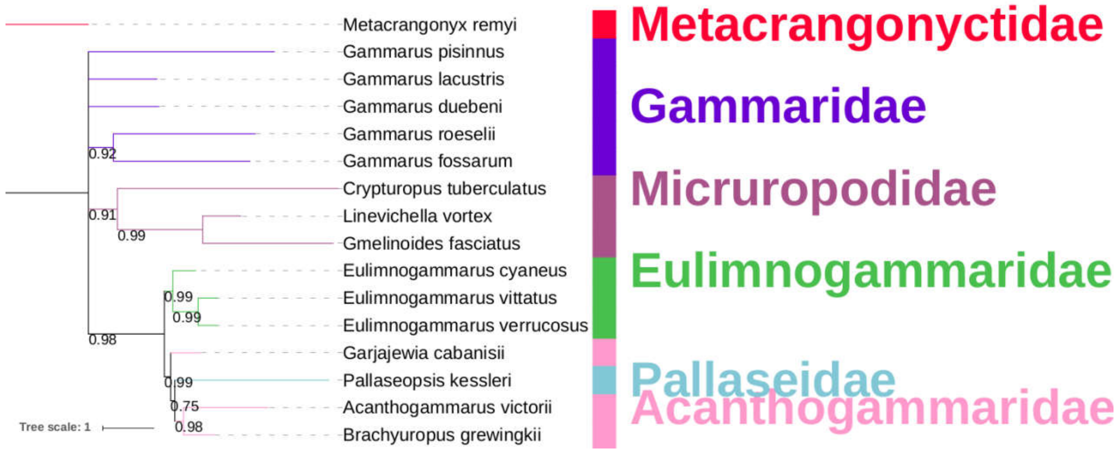

PB_PCGRT_NUC_Gammaroidea analysis. Phylogram was inferred using the 'heterogeneous' CAT-GTR model implemented in PhyloBayes. Dataset comprised nucleotide sequences of all genes (PCG+rRNA+tRNA) of the Gammaroidea dataset. *Metacrangonyx remyi* is the outgroup (branch cropped). Scale bar corresponds to the estimated number of substitutions per site. Posterior probability support values <1.0 are shown next to nodes. GenBank accession numbers are available in the Additional file 1. Family names and corresponding coloured bars are shown to the right.

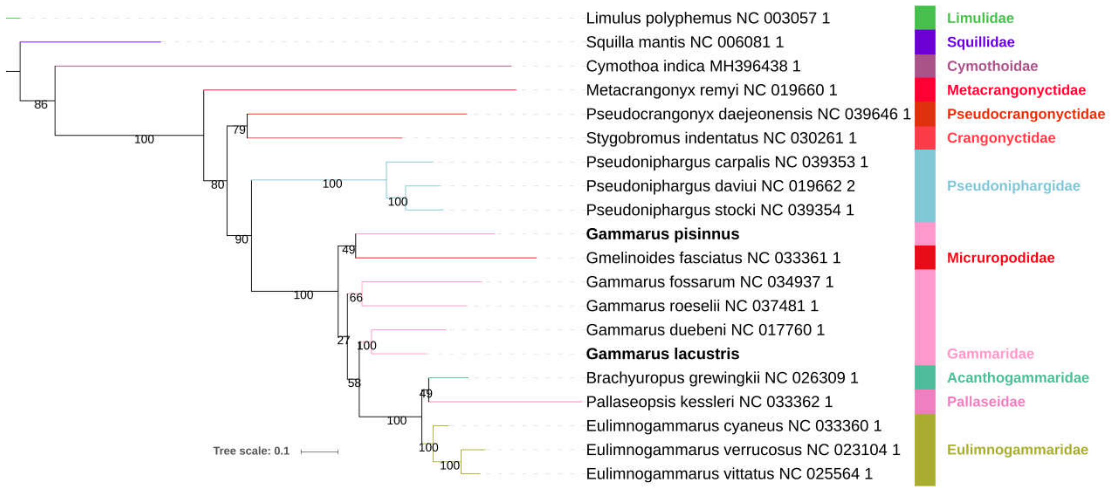

ML_PCG_AA_Part_Senticaudata analysis. Phylogram was inferred via a Maximum Likelihood analysis of concatenated (partitioned) amino acid sequences of all 13 protein-coding genes of the Senticaudata dataset. *Limulus polyphemus* (branch cropped), *Squilla mantis* and *Cymothoa indica* are outgroups. Scale bar corresponds to the estimated number of substitutions per site. Bootstrap support values are shown next to nodes, and GenBank (RefSeq) accession numbers next to species names. Family names and corresponding coloured bars are shown to the right. The two newly-sequenced species are bolded, and branch colours correspond to families.

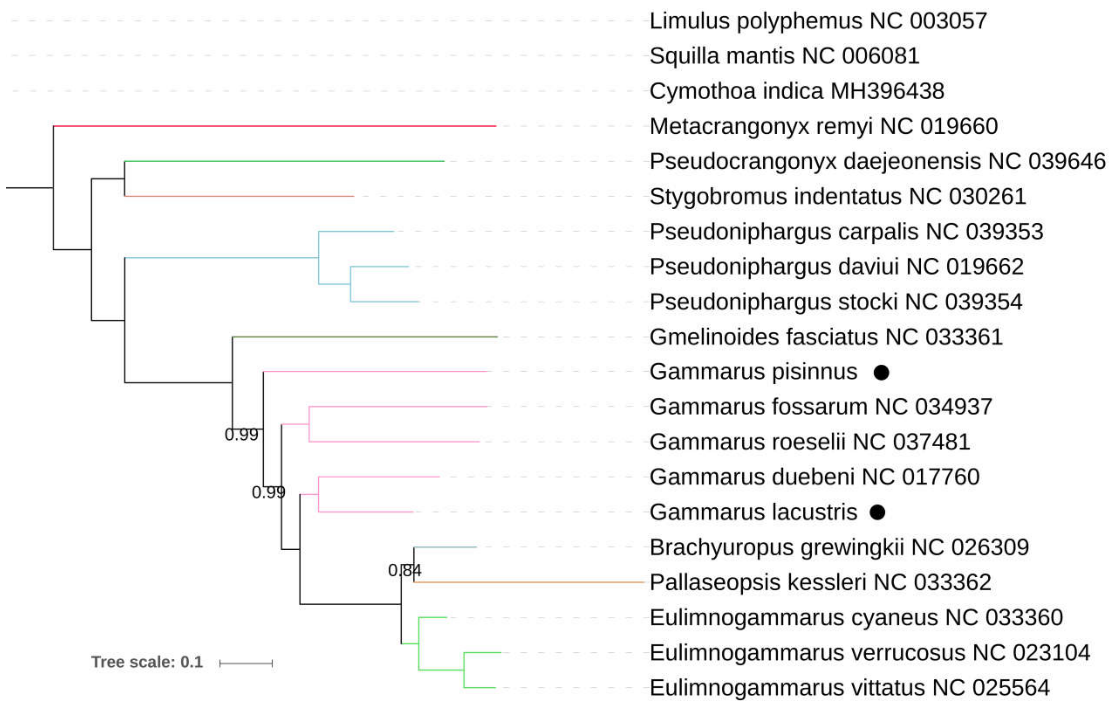

BI_PCG_AA_Part_Senticaudata analysis. Phylogram was inferred via a Bayesian Inference analysis of the concatenated (partitioned) dataset comprising amino acid sequences of all 13 protein-coding genes of the Senticaudata dataset. *Limulus polyphemus*, *Squilla mantis* and *Cymothoa indica* are outgroups (branches cropped). Scale bar corresponds to the estimated number of substitutions per site. Posterior probability support values <1.0 are shown next to nodes, and GenBank (RefSeq) accession numbers next to species names. The two newly-sequenced species highlighted with a black dot, and branch colours correspond to families.
