# Supplementary material for: Disentangling the interplay of positive and negative selection forces that shaped mitochondrial genomes of Gammarus pisinnus and Gammarus lacustris
Source: R Soc Open Sci. 2020 Jan 8;7(1):190669. doi: 10.1098/rsos.190669 (PMC7029888; doi:10.1098/rsos.190669)
Supplement: Additional file 3. Primers used for the amplification and sequencing of the mitochondrial genomes of Gammarus pisinnus and Gammarus lacustris. [file rsos190669supp3.docx]

**Additional file 3. Primers used for the amplification and sequencing of the mitochondrial genomes of *Gammarus pisinnus* and *Gammarus lacustris*.**

*Gammarus pisinnus*

| **Fragment**  **No.** | **Gene or**  **region** | **Primer**  **name** | **Sequence (5’-3’)** | **Length**  **(bp)** |
| --- | --- | --- | --- | --- |
| F1 | *ND2* | XAF1 | GATTTTTAGCTTGACTTGG | 698 |
|  |  | XAR1 | AGTTAACTGGGTAATGAC |  |
| F2 | *ND2-C0X1* | XAF2 | GTACTAATTTCTCTTTTGGTGC | 937 |
|  |  | XAR2 | GCAAGATCCACAGCTGCTCC |  |
| F3 | *COX1* | XAF3 | GATTATTACCTCCTTCTCTAAC | 968 |
|  |  | XAR3 | GAAAGTTAGGTTAACACCTAC |  |
| F4 | *COX1-COX2* | XAF4 | GTAGTAGCTCACTTTCATTATG | 926 |
|  |  | XAR4 | GAGAATTAACCGGTAAGAC |  |
| F5 | *COX2* | XAF5 | GATATTGATCCTATGAATAC | 370 |
|  |  | XAR5 | GAATAATAGGATATCACTCAG |  |
| F6 | *COX2-ATP6* | XAF6 | CGTTCCAGGCCGTTTGAACC | 911 |
|  |  | XAR6 | GCAGTTAAGCGTATGGTTAGTG |  |
| F7 | *ATP6-ND5* | XAF7 | CAACTAGTCTTTACCGCTG | 2600 |
|  |  | XAR7 | CGCTATAAGAAGGCCTGCTC |  |
| F8 | *ND5-CYTB* | XAF8 | CTACACCAACAACTCTTATC | 4236 |
|  |  | XAR8 | GTAGTTGAGGAGTCAGCAGG |  |
| F9 | *CYTB-16S* | XAF9 | GCCCATATTACTTTCTTACACC | 1917 |
|  |  | XAR9 | GGTTAAGTTACTCTAGGG |  |
| F10 | *16S* | XAF10 | GGTTGAACAAACCTTCTAC | 387 |
|  |  | XAR10 | CTGTGCTAAGGTAGCATAATC |  |
| F11 | *16S-12S* | XAF11 | GCTTCTAGGGTCTTATCGTC | 912 |
|  |  | XAR11 | GATAGTTAAGACAAGTAGTC |  |
| F12 | *12S* | XAF12 | CGACTTATCCCTAAGAGATTG | 374 |
|  |  | XAR12 | GTCAGGTCAATTAGAACCAGG |  |
| F13 | *12S-*CR | XAF13 | AACTAGCGAGGCTTATCG | 575 |
|  |  | XAR13 | CGAGCAACATAAGATGTGAG |  |
| F14 | CR*-ND2* | XAF14 | TCTAGCCCTTTACTTCTAC | 2376 |
|  |  | XAR14 | CACTTAAGAAGTAAAGCTCTAC |  |

Length is the amplicon length, CR is control region.

*Gammarus lacustris*

| **Fragment**  **No.** | **Gene or**  **region** | **Primer**  **name** | **Sequence (5’-3’)** | **Length**  **(bp)** |
| --- | --- | --- | --- | --- |
| F1 | *COX1* | XZF1 | GTTCGGCAACTGACTAGTTCC | 1194 |
|  |  | XZR1 | GCTTCTATGATTATGAGCAC |  |
| F2 | *COX1-COX2* | XZF2 | GCAAAGACACATTTCTATG | 809 |
|  |  | XZR2 | GGATAGGCAGAGCCAGCCTG |  |
| F3 | *COX2* | XZF3 | GGCACCAATGATACTGGTCC | 296 |
|  |  | XZR3 | GCAGATTTCTCTGCATTGTC |  |
| F4 | *COX2-ATP6* | XZF4 | GGCTCTGCCTATCCACTCTC | 943 |
|  |  | XZR4 | GGGTATGAGTAGAGCAGGTG |  |
| F5 | *ATP6-COX3* | XZF5 | GCTGGCTTAGCCCCTTACG | 859 |
|  |  | XZR5 | CATGTAACTGTGACACCAG |  |
| F6 | *COX3-ND5* | XZF6 | CTGAGCTTTCTTTCACAGTAG | 1492 |
|  |  | XZR6 | CTTCAGAAAAGAAGTATACC |  |
| F7 | *ND5* | XZF7 | CAAACCAGCCAAGGTCCA | 568 |
|  |  | XZR7 | GCAATAGGTGTAGTGTTTGG |  |
| F8 | *ND5-ND4* | XZF8 | CCAATAATCAAACAAAGTCC | 1422 |
|  |  | XZR8 | GAGTCTAGTTAGATGAAGAAG |  |
| F9 | *ND4* | XZF9 | GACCTCAATGAGCAGCACAG | 904 |
|  |  | XZR9 | GGTTGGGGATACCAGCCTGAG |  |
| F10 | *ND4-ND6* | XZF10 | CTCTATTTAGCACGCCTAAATC | 1353 |
|  |  | XZR10 | CAAGAAGATTGTGGTTGCTAG |  |
| F11 | *ND6-ND1* | XZF11 | TCCCCGTACTTGTTACTAATC | 1749 |
|  |  | XZR11 | CACAGAGTATGGGTCAGGTG |  |
| F12 | *ND1* | XZF12 | CCCAAAAACCACTATCATTGG | 558 |
|  |  | XZR12 | GTAGTCAGTTTGACACTATC |  |
| F13 | *ND1-16S* | XZF13 | CGTAGCCTCCCTAGCATAGAG | 711 |
|  |  | XZR13 | GTTACTCTAGGGGTAACAGTGC |  |
| F14 | *16S* | XZF14 | GATAAAATCCAATCTGGCTC | 497 |
|  |  | XZR14 | GGTGTAAGTAAGATAAGACC |  |
| F15 | *16S-12S* | XZF15 | CAAAGAATTCTTACCTCGAC | 850 |
|  |  | XZR15 | GCTAGATCAAGGTGCAGAGTG |  |
| F16 | *12S* | XZF16 | GTTTCGACTTATCTCTTAGAG | 445 |
|  |  | XZR16 | GCTATCTTTTGTTAGAACGAG |  |
| F17 | *12S-COX1* | XZF17 | CAAGCAAACTCCTCTAAATCAG | 3465 |
|  |  | XZR17 | TCCGCTCTCCACTAGACCTC |  |

Length is the amplicon length.
